# Bioavailability of the Common Cold Medicines in Jellies for Oral Administration

**DOI:** 10.3390/pharmaceutics12111073

**Published:** 2020-11-10

**Authors:** Ki Hyun Kim, Minju Jun, Mi-Kyung Lee

**Affiliations:** 1 CKD Research Institute, Gyeonggi 16995, Korea; hyun@ckdpharm.com (K.H.K.); minju1@ckdpharm.com (M.J.); 2 Department of Pharmaceutical Sciences, Woosuk University, Jeonbuk 55338, Korea

**Keywords:** jelly, bioavailability, acetaminophen, chlorpheniramine, dextromethorphan, methylephedrine

## Abstract

Jellies for oral administration have been suggested as alternative dosage forms to conventional tablets for improved palatability and compliances for pediatric and geriatric patients. To evaluate the effect of jelly formulation on the bioavailability of cold medicines, two types of jellies were prepared for a fixed-dose combination of acetaminophen (AAP), chlorpheniramine maleate (CPM), dextromethorphan hydrobromide (DMH), and dl-methylephedrine hydrochloride (MEH). Jelly-S and Jelly-H were fabricated using carrageenan and locust bean gum in the absence and presence of xanthan gum, respectively. In vitro dissolution and in vivo absorption of the four drugs in the jellies were compared with other conventional formulations, a syrup and two types of immediate-release (IR) tablets with different hardness, Tablet-S (15 kPa) and Tablet-H (20 kPa). All the formulations exhibited more than 80% dissolution rate within 2 h even though the syrup, Jelly-S, and Tablet-S showed higher 30-min dissolution compared to Jelly-H and Tablet-H. The dissolution rates from the jellies decreased with increasing pH, which resulted in the slowest dissolution in pH 6.8 compared to the syrup and IR tablets. When administered orally to beagle dogs, all five formulations were determined not to be bioequivalent. However, Jelly-S and Jelly-H showed 0.82–1.05 of the geometric mean ratios (GMRs) of AUC_0-t_ for all four drugs compared to the syrup suggesting comparable absorption. In two IR tablets, GMRs of AUC_0-t_ were in a range of 0.55–0.95 indicating a tendency of lower absorption than the syrup and jellies. In conclusion, jelly can be a patient-centered formulation with comparable bioavailability to syrup.

## 1. Introduction

The common cold, an acute upper respiratory viral infection, is a mild illness that typically presents with nasal congestion, rhinorrhea, sour throat, cough, headache, and fever. These symptoms are generally self-limited. Hence, people usually recover in seven or ten days [1]. The primary goal of the treatment is a reduction in duration and severity of the symptoms by non-pharmacological and pharmacological interventions. As pharmacological interventions, analgesics, antihistamines, and decongestants have been applied as mono- and combination therapy [2]. Variable pharmaceutical products included acetaminophen (AAP), chlorpheniramine maleate (CPM), dextromethorphan hydrobromide (DMH), and dl-methylephedrine hydrochloride (MEH) in combination therapy for the common cold.

AAP is the most widely used non-prescription analgesic globally and is considered safe in therapeutic doses producing negligible therapeutically significant drug interaction even though overdose can cause hepatotoxicity [3,4]. AAP is categorized as Biopharmaceutical Classification System (BCS) class I, which is highly soluble and highly permeable [5,6,7,8]. The absorption of BCS class I drugs can be influenced mainly by gastric emptying rate rather than dissolution or formulation parameters unless the dissolution rate is slower than gastric emptying time [9]. AAP is rapidly and almost completely absorbed, mainly in the small intestine, with negligible absorption in the stomach [10]. CPM is an antihistamine and used widely for symptomatic relief of the common cold and allergic conditions [11]. CPM is also categorized into BCS class I [12]. However, its absorption has been reported to be sensitive to local gastrointestinal conditions and formulation employed [13,14,15]. The site of absorption for CPM is thought to be the small intestine due to its basicity [11,16]. DMH is an antitussive agent included commonly in cough and cold preparations [17]. Because it has high water solubility and short half, there have been various attempts to develop sustained-release preparations [18]. DMH belongs to the BCS class II, which exhibits low solubility and high permeability, suggesting its dissolution and absorption can be significantly affected by formulation factors [19]. Methylephedrine is one of the ephedra alkaloids and indicated in the treatment of allergy, asthma, cold, cough in flu, and cold in many countries, even though MEH is not legally available in the USA due to the possibility of abuse [20]. MEH’s little or no physical dependence is evident because only a small amount of MEH is included in cold preparations and used for a short period [21].

Conventional solid dosage forms such as tablets and capsules have been the most widely applied formulations. However, alternative formulations have been suggested to improve the patient’s acceptance of the therapy [22,23,24]. Apart from syrup which is preferred for pediatric patients, there have been various novel formulations such as orodispersible or multipartculate preparations and mini-tablets which can be easily swallowed [25]. These alternative dosage forms can help geriatric patients who also suffer from a reduced ability to swallow [26]. Jellies for oral administration can also be preferred dosage forms for pediatric and elderly patients [27]. According to the Korean Pharmacopeia 11th edition (KP XI), jellies for oral administration are described as non-flowing gel-like preparations having a particular shape, and they can be prepared using various natural gums or other hydrophilic polymers [28]. Additionally, jelly does not require water for swallowing in contrast to tablets and capsules because it is soft and moist. Similar to syrup, taste masking is possible using sweetening and flavoring agents. Harada et al. developed oral jelly formulations containing donepezil hydrochloride to use for Alzheimer’s disease [29]. Even though there has been increased demand for the jelly formulations, few studies reported the in vitro and in vivo performance of oral jellies.

In the present study, we developed two types of jelly formulations, Jelly-S and Jelly-H, using carrageenan and locust bean gum in the absence and presence of xanthan gum, respectively. Furthermore, we compared bioavailabilities of the drugs in the jellies with those in other conventional dosage forms such as syrup and immediate-release tablets.

## 2. Materials and Methods

### 2.1. Materials

AAP and DMH were supplied by Lianyungang Kangle (Lianyungang, China) and Wockhardt (Mumbai, India), respectively. CPM and MEH were provided by Chemtros (Ansan, Korea). Kappa and iota carrageenan were obtained from Dupont Nutrition (Wilmington, DE, USA). Xanthan gum and locust bean gum were from CP Kelco (Atlanta, GA, USA). The orange flavor was supplied by Bolak (Hwaseong, Korea). A commercially available syrup formulation, Coldy^®^ S (Samil Pharm. Co., Seoul, Korea), was purchased as an over-the-counter drug product. According to the information provided on the website of the Korean Ministry of Food and Drug Safety (https://nedrug.mfds.go.kr), 100 mL of Coldy^®^ S syrup contains 1000 mg AAP, 12.5 mg CPM, 75 mg DMH, and 125 mg MEH with excipients such as sucrose, hydroxyethylcellulose, povidone, d-sorbitol, methyl paraben, strawberry flavor, and water. The sources of the excipients for immediate-release (IR) tablets were as follows: microcrystalline celluloses (Ceolus^®^ PH-101 and Ceolus^®^ PH-102, Asahi Kasei, Nobeoka, Japan), lactose hydrate (SuperTab^®^ 11SD, DFE pharma, Norten-Hardenberg, Germany), low substituted hydroxypropylcellulose (L-HPC LH-11, Shin-Etsu Chemical, Joetsu, Japan), hydroxypropylcellulose (HPC-L, Nippon Soda, Tokyo, Japan), colloidal silicon dioxide (Aeroperl 300, Evonik, Essen, Germany), glyceryl behenate (Compritol 888 ATO, Gattefosse, Saint-Priest, France), magnesium stearate (Magnesium stearate (vegetable-based), Faci Asia Pacific, Jurong Island, Singapore), and Opadry^®^ (03B28796 White, Colorcon, Harleysville, PA, USA). For dissolution media, extra pure sodium chloride (Daejung Chemicals catalog number 7548-4400, purity >99.0%, Gyeonggi-Do, Korea), sodium acetate trihydrate (Samchun Chemicals catalog number S0274, purity ≥98.5%, Seoul, Korea), and potassium phosphate monobasic (Daejung catalog number 6613-4400, purity >99.0%, Gyeonggi-Do, Korea) were used. Methanol and acetonitrile for HPLC were purchased from Burdick & Jackson (Muskegon, MI, USA), while formic acid from J & K scientific (Beijing, China). All other materials were of reagent grade and used without further purification.

### 2.2. Preparation of Oral Jelly

Two types of oral jellies with different gel strength were prepared, as shown in Table 1. Briefly, carrageenan, locust bean gum, and xanthan gum were dissolved in purified water and then mixed with an aqueous solution containing drugs, preservative, flavoring agent, and sweetener at the elevated temperature (85 °C). The final mixture was filled into a stick-type package (40 × 150 mm) and incubated at room temperature to obtain jelly. The strength of the jelly was measured using a texture analyzer, TA-XTplusC, equipped with a 35 mm round probe (Stable Micro Systems, Godalming, UK) in accordance with the Physical Testing of Jelly described in Food Code of Republic of Korea with a slight modification [30]. Briefly, the jelly was cut into a piece of 10 (length) × 10 mm (width) in size and compressed at a pre-test speed of 5 mm/s, test speed of 2 mm/s, and post-test speed of 2 mm/s with a pressure up to 98% compression. The strength was determined as the force at the first peak from the test force versus time curve. The measured gel strengths were within 3–4 N for Jelly-S and 8–9 N for Jelly-H, which were the ranges to be easily crushed or broken within the mouth.

### 2.3. Preparation of IR Tablets

The IR tablets were prepared by indirect compression after wet-granulation as follows using the composition listed in Table 2. First, the following drugs and excipients were mixed for 3 min at 200 rpm: AAP, CPM, DMH, MEH, microcrystalline cellulose (PH-101), hydroxypropyl cellulose (HPC), colloidal silicon dioxide, and glyceryl behenate. The wet granulation was carried out by adding binder solution (HPC in water) in a high-shear granulator (NMG-10L, Nara Machinery, Tokyo, Japan) with an impeller speed of 200 rpm, chopper speed of 1800 rpm, and 4 min of granulation time. The wet granules were sieved with a conical mill (Comil U3, Quadro Engineering, Waterloo, ON, Canada) equipped with a 4 mm sieve and then dried in an oven at 60 °C until the designated moisture content (within 2%) was achieved. The dried granules were sieved with a conical mill equipped with a 1.2 mm sieve and mixed with lactose hydrate, microcrystalline cellulose (PH-102), and low substituted HPC. The final mixture was lubricated by adding and mixing with magnesium stearate sieved through 30 mesh. Finally, the lubricated mixture was pressed using a single punch tablet press (XP 1, Korsch AG, Berlin, Germany) with an 18 mm × 9 mm oblong-shaped punch. The tablets were coated with Opadry^®^ (03B28796, White) using a tablet coater (HC-LABO, Freund Corporation, Tokyo, Japan). The resulting tablets had a hardness of 15 (Tablet-S) or 20 kP (Tablet-H).

### 2.4. Determination of Drug Content in the Formulations

Drug contents in each formulation were determined with the method described in the Korea Pharmacopoea XI with slight modifications as follows using a Shiseido Nanospace SI 2 HPLC system (Osaka, Japan). Five packages of jellies, syrup, or tablets were added into a 1000-mL volumetric flask with water to mix for three hours with continuous stirring. After dilution with pH 1.2 media by 5-folds, the solution was filtered through 0.45 μm. An amount of 100 μL of the filtered solution was injected onto the HPLC column (Intertsil ODS-4, 4.6 *×* 250 mm, 5 μm) and separated with gradient elution of mobile A (a mixture of 0.5 M hyperchloric acid, tetrahydrofuran, and acetic acid at a ratio of 850:150:10) and mobile B (a mixture of mobile A and acetonitrile at a ratio of 10:90); 100% mobile A for 14 min, 70% mobile A and 30% mobile B from 14 min to 22 min, 100% mobile A from 22 min to 25 min. The flow rate was 1 mL/minute. CPM and MEH were detected at 254 nm, while AAP and DMH were determined at 280 nm. In addition, drug content was re-determined within 3 days before administration to beagle dogs to ensure drug content and stability during delivery to the animal study facility.

### 2.5. Dissolution Test

Comparative in vitro dissolution tests were performed in 900 mL of water, pH 1.2, pH 4.0, and pH 6.8 media in accordance with the guidelines for the bioequivalence test published by the Korean Ministry of Food and Drug Safety. The dissolution test was carried out using the paddle method with a stirring rate of 50 rpm at 37 °C. The pH 1.2 medium was prepared by adding 7.0 mL of concentrated hydrochloride solution into water, dissolving 2.0 g of sodium chloride, and then adjusting into 1000 mL with water. Acetate buffer, pH 4.0, was prepared by mixing 41 parts of 0.05 M acetic acid with 9 parts of 0.05 M sodium acetate solution and adjusting the pH to 4.0 using 0.2 M sodium hydroxide or hydrochloride solution. Phosphate buffer, pH 6.8, was prepared by mixing 250 mL of 0.2 M potassium dihydrogen phosphate solution with 118 mL of 0.2 M sodium hydroxide solution. An amount of 5 mL of the dissolution medium was taken at the time points of 5, 10, 15, 30, 45, 60, 90, and 120 min, filtered, and analyzed by high performance liquid chromatography as listed in the Korean Pharmacopeia 11th edition using the detection wavelength of 280 nm for AAP, 280 nm for DMH, 254 nm for CPM, and 254 nm for MEH. The details for the HPLC analysis conditions were the same with those used in the drug content determination. For comparison of dissolution profiles, model-independent similarity factor (*f_2_*) for every pair was calculated as follows:

(1) $$f_{2}=50\;\times \log\left\{{\left[1+\frac{1}{n}\overset{n}{\underset{j=1}{\sum }}{\left(R_{j}-T_{j}\right)}^{2}\right]}^{-0.5}\times 100\right\}$$

where *n* is the number of time points and *R_j_* and *T_j_* are the percentages of reference and test product, respectively, released into dissolution medium at time *j*. According to the FDA guidance, dissolution profiles are similar if *f_2_* values are between 50 and 100 [31]

### 2.6. Pharmacokinetic Study in Beagle Dogs

A pharmacokinetic study was performed in beagle dogs after oral administration in Medicilon Preclinical Research (Shanghai) LLC (Shanghai, China). The beagle dogs were male and 1.6 ± 0.3 years old. The average body weight was 10.5 ± 1.7 kg. The study was performed by 5 × 5 cross-over design. Dogs were fasted for 12 h prior to the administration. Fifteen dogs were randomly assigned into 5 groups (3 animals per each group), and each group was treated orally with Jelly-S, Jelly-H, Tablet-S, Tablet-S, or Coldy^®^ S Syrup. After one-week wash-out time, each group received a different formulation at each period. The administered doses were 150 mg as AAP, 1.875 mg as CPM, 11.25 mg as DMH, and 18.75 mg as MEH per a dog. One pack (15 g) of jelly was administered as a whole using a 50-mL syringe. The syrup (15 mL) was given using a 20-mL syringe. A whole tablet was allowed to be swallowed by administering 20 mL of purified water immediately. Blood samples were collected at 0.167, 0.33, 0.5, 0.75, 1, 2, 4, 6, 12, and 24 h after administration. Blood samples were placed into tubes containing K_2_-EDTA and centrifuged at 2200× *g* for 10 min at 2–8 °C to separate plasma. The plasma samples were stored at −80 °C until analysis. All animal studies were performed according to the guidelines of the Ethics Committee for the use of Experimental Animals and approved by the Institutional Animal Care and Use Committee of Medicilon Preclinical Research (Approval ID 03010-20002 on 9 March 2020).

### 2.7. LC/MS/MS Analysis of Drugs in Plasma

The plasma concentration was determined by LC/MS/MS. Liquid chromatography (LC) system was Shimadzu (Osaka, Japan) UPLC system equipped with two pumps (LC-30AD), a degasser (DGU-20A3R, DGU-20A5R), an autosampler (SIL-30AC), and a column oven (CTO-20A). Mass spectrometric analysis was performed using a TQ 5500 (triple-quadrupole) instrument from Applied Biosystems/MDS Sciex with an ESI Ionsource. The data acquisition and control system was created using Analyst 1.6.3 Software (AB Sciex Pte. Ltd., Estate, Singapore, 2015) from Applied Biosystems/MDS Sciex. Plasma samples (0.02 mL) were mixed with 200 μL of internal standard (10 ng/mL verapamil in acetonitrile) and centrifuged for 7 min at 18,000× *g*. An aliquot (120 μL) of supernatant was transferred to a 96-well plate and mixed with 120 μL of water before injection onto the column. The acceptance criteria for calibration curves were as follows. Minimum six non-zero standards must be within 15% relative standard error (RSD) of nominal value, within 20% RSD at lower limit of quantitation. The correlation coefficient (*r*^2^) of the calibration curve should be ≥0.985. Accuracy, precision, and other validation parameters met the FDA guidance for industry (Bioanalytical Method Validation, issued in May 2018). The drugs were separated through the CORTECS T3 column (2.7 µm, 2.1 × 100 mm, Waters, Milford, MA, USA) at 40 °C of column oven temperature by gradient elution of 0.1% formic acid in 10 mM ammonium formate (mobile phase A) and 0.1% formic acid in methanol (mobile phase B) at a flow rate of 600 µL/min. The elution program for AAP was 0–0.02 min (1% B), 0.02–0.08 min (82% B), 0.80–1.30 min (82% B), 1.30–1.31 min (1% B), and 1.31–2.00 min (1% B). For CPM, DMH, and MEH, the elution program was 0–0.01 min (20% B), 0.01–0.03 min (40% B), 0.03–0.50 min (40% B), 0.50–0.90 min (86% B), 0.90–1.40 min (86% B), 1.40–1.41 min (20% B), and 1.41–2.00 min (20% B). The injection volume was 8 µL. The MS detection was performed with positive MRM an ESI ionization mode. The analytical method was applied after validation according to the Guidance for Industry: Bioanalytical Method and Validation by FDA.

### 2.8. Pharmacokinetic Data and Statistical Analysis

Pharmacokinetic parameters were calculated by non-compartmental analysis using Pheonix WinNonlin (ver 8.1, Pharsight-A Certara Company, Princeton, NJ, USA). The maximum plasma concentration (C_max_) and the time to reach C_max_ (T_max_) were obtained directly from the time-concentration curves. The area under the curve from zero to the last sampling time (AUC_0-t_) was calculated using the linear trapezoidal rule. The AUC from zero to infinity (AUC_0-__∞_) was determined by adding C_last_/k to the AUC_0-t_, where C_last_ is the last measured plasma concentration and k the terminal elimination constant. The terminal elimination constant (k) was obtained by the linear regression of the terminal data points in the time-log concentration curve. After logarithmical transformation of AUC_0-t_ and C_max_, geometric mean ratios (GMR) and 90% confidence intervals (CI) were estimated using the syrup as reference formulation to evaluate bioequivalence among formulations. In addition, the significance of AUC and C_max_ among formulations was assessed using one-way ANOVA for repeated measures with Turkey’s multiple comparison test. T_max_ values were compared using the non-parametric Kruskal–Wallis test followed by Dunn’s multiple comparison test. Differences were considered significant at *p* < 0.05.

## 3. Results

### 3.1. Drug Contents in the Formulations

Drug contents in the syrup, jellies, and IR tablets were determined using HPLC and shown in Table 3. The content was expressed as % nominal content. The contents for AAP, CPM, DMH, and MEH were within 98.0–102.0% in all five formulations, and there was no statistically significant difference. In addition, the contents were assessed within 3 days before administration to beagle dogs to ensure the drug content and stability.

### 3.2. In Vitro Dissolution in Various pH Media

As mentioned above, the jellies with different gel strengths were obtained in the absence or presence of xanthan gum: 3–4 N for Jelly-S without xanthan gum and 8–9 N for Jelly-H with xanthan gum. By using different amounts of magnesium stearate, the hardness of tablets was controlled to 15 (Tablet-S) or 20 kPa (Tablet-H) to show slightly altered disintegration and dissolution.

**Figure 1:**
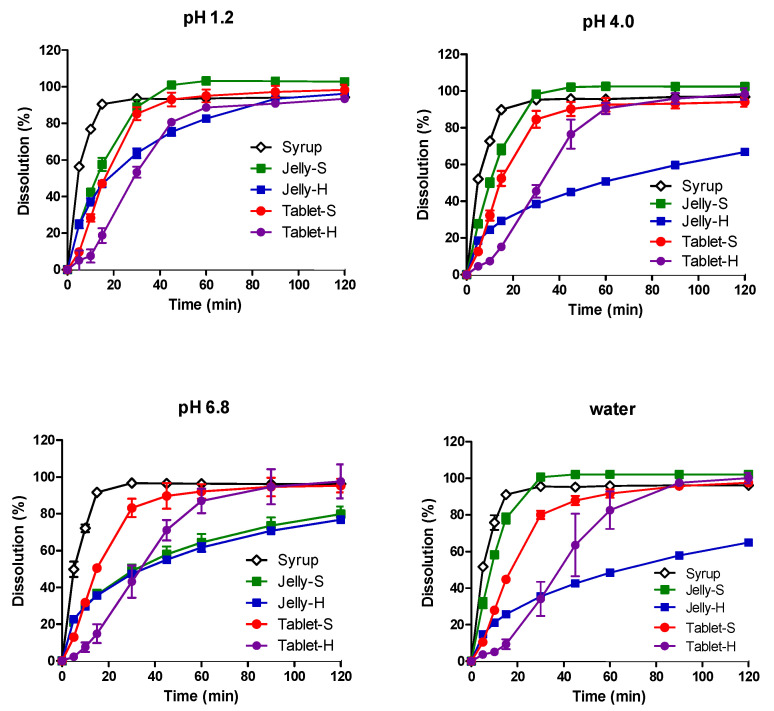
Dissolution profiles of acetaminophen from various formulations in pH 1.2, 4.0, 6.8, and water at 37 °C. Each point represents mean ± standard deviation (*n* = 6).

**Figure 2:**
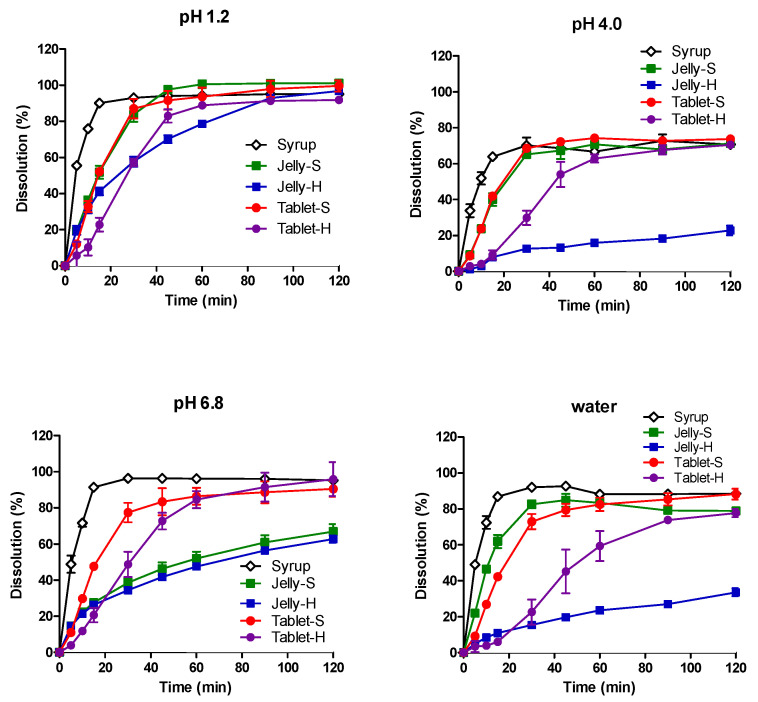
Dissolution profiles of chlorpheniramine maleate from various formulations in pH 1.2, 4.0, 6.8, and water at 37 °C. Each point represents mean ± standard deviation (*n* = 6).

**Figure 3:**
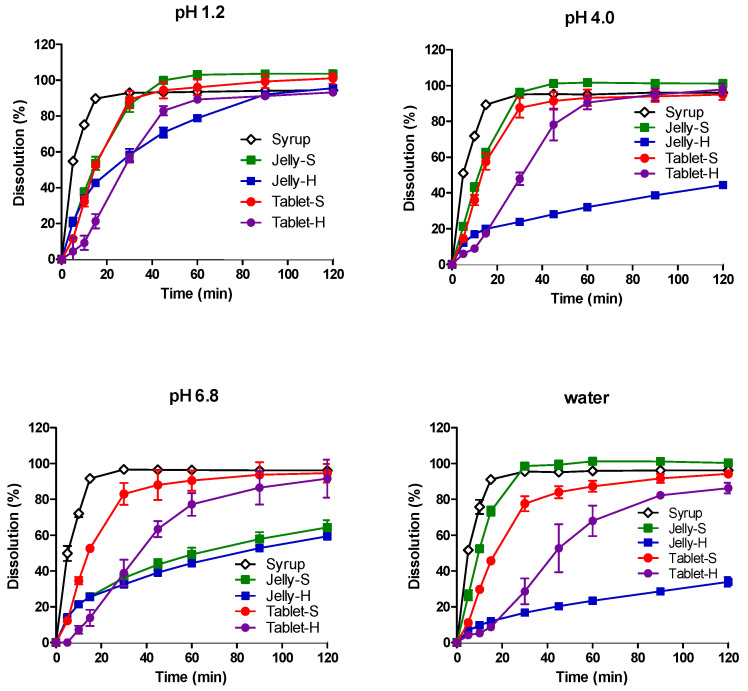
Dissolution profiles of dextromethorphan hydrobromide from various formulations in pH 1.2, 4.0, 6.8, and water at 37 °C. Each point represents mean ± standard deviation (*n* = 6).

**Figure 4:**
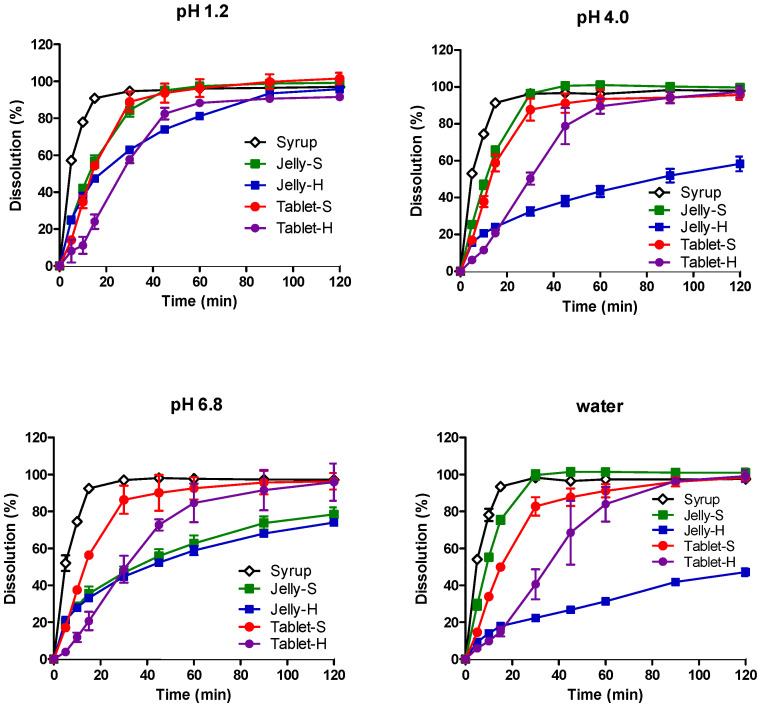
Dissolution profiles of dl-methyl ephedrine hydrochloride from various formulations in pH 1.2, 4.0, 6.8, and water at 37 °C. Each point represents mean ± standard deviation (*n* = 6).

The dissolution profiles of AAP, CPM, MPH, and DMH from the five formulations in water, pH 1.2, pH 4.0, and pH 6.8 media are shown in Figure 1, Figure 2, Figure 3 and Figure 4, respectively. In pH 1.2 media, the dissolutions of all four drugs from the syrup were rapid, showing higher than 90% dissolution within 30 min. Compared to the syrup, the dissolutions of the drugs from the jellies and IR tablets were slightly retarded as expected, exhibiting much slower dissolution from Jelly-H and Tablet-H than the other three formulations. Meanwhile, higher pH reduced the dissolution rates from the Jelly-S, Jelly-H, and Tablet-H, showing less than 50% dissolution in 30 min in pH 6.8 media. Even after 2 h, Jelly-S and Jelly-H released less than 80% of the drugs in pH 6.8, while Tablet-H showed higher than 90% dissolution similar to the Syrup and Tablet-S formulations. Nevertheless, the decreased dissolution in pH 6.8 was not likely to reduce the absorption significantly because most drugs were already dissolved in the stomach. However, the effect of the reduced dissolution on the absorption should be addressed in the in vivo absorption study. Hence, pharmacokinetic study for the various formulations was performed using beagle dogs in the present study.

Jelly-S and Jelly-H formulations contained carrageenan, which is known to be hydrolyzed in acidic pH [32,33]. Hence, the increased dissolution of the drugs from Jelly-S and Jelly-H in acidic pH was likely due to acidic hydrolysis of carrageenan. However, the increased dissolution by acidic pH was alleviated by the addition of Xanthan gum into the formulation, as shown in Xanthan gum-containing Jelly-H compared to the non-Xanthan gum formulation, Jelly-S. For Jelly-S, the dissolutions of the drugs were increased by decreasing pH in order of pH 1.2 > pH 4.0 > pH 6.8. On the contrary, Jelly-H showed increased dissolution in pH 1.2, while similar low dissolution rate between pH 4.0 and 6.8. The different behavior between Jelly-S and Jelly-H was likely due to the enhanced gel strength by xanthan gum. In purified water, Jelly-S showed a higher dissolution rate compared to Jelly-H. Similarly, Tablet-S exhibited higher dissolution in water than Tablet-H did. The dissolution pattern in the salt-free condition appeared to offer information on the effect of gel strength of the jellies or tablet strength on the hydration rate, which can subsequently affect dissolution rate. As expected, higher gel strength and tablet hardness caused lower dissolution rates regardless of the types of drugs, as shown in Figure 1, Figure 2, Figure 3 and Figure 4.

To compare dissolution profiles, the similarity factor (*f_2_*) was calculated. Every possible pair presented *f_2_* values less than 50 except Jelly-S and Jelly-H in pH 6.8, suggesting that the five formulations were not equivalent in the in vitro dissolution [31].

### 3.3. In Vivo Pharmacokinetics

**Figure 5:**
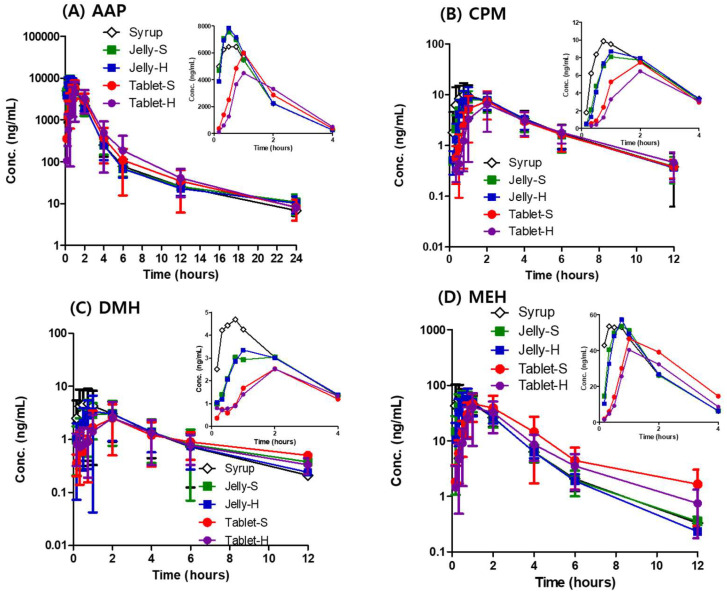
Plasma concentration-time profile of (**A**) AAP, (**B**) CPM, (**C**) DMH, and (**D**) MEH in dogs after oral administration of various formulations at a dose of 150, 1.875, 18.75, and 11.25 mg, respectively. Each point represents mean ± standard deviation (*n* = 15).

The plasma concentration profiles of AAP, CPM, DMH, and MEH are presented in Figure 5. As shown in the initial plasma concentration profile for 4 h, the jellies and syrup showed rapid absorption of AAP, while both tablets retarded the absorption of AAP exhibiting lower C_max_ and delayed T_max_ (Table 4). As mentioned in the dissolution study, the delayed dissolution of AAP from the Jelly-H in pH 4.0, 6.8, and water did not affect in vivo absorption because most AAP was dissolved in the stomach to be absorbed in the small intestine. Similarly, Tablet-H delayed the absorption of the other three drugs as well. Tablet-H reduced the mean C_max_ of CPM by approximately 30% compared to the syrup (6.9 ± 4.3 vs. 11.3 ± 6.3) even though the difference was not statistically significant. On the contrary, Jelly-S and Jelly-H showed comparable C_max_ and T_max_ of CPM to the syrup as shown in Table 3. Compared to the syrup, the absorption of DMH was delayed more by the jellies and tablets. Jelly-S and Jelly-H reduced C_max_ of DMH by approximately 30% compared to the syrup, while Tablet-S and Tablet-S decreased the C_max_ by more than 50%. For MEH, a similar pattern was observed: more reduced C_max_ and further delayed T_max_ by Tablet-S and Tablet-H compared to the Jelly-S and Jelly-H. Overall, the extent and rate of the absorption of the drugs were in the order of the Syrup > Jelly-S > Jelly-H > Tablet-S > Tablet-H. Even though Jelly-H exhibited the slowest dissolution in pH 1.2, 4.0, 6.8, and water, and five formulations were not bioequivalent, the in vivo absorption of AAP and MEH formulated in Jelly-H was almost comparable to those in the syrup.

Point estimates and 90% confidence intervals for AUC_0-t_ and C_max_ are presented in Table 5, Table 6, Table 7 and Table 8 for AAP, CPM, DMH, and MEH, respectively. The 90% confidence intervals for the mean ratios of each formulation to the syrup were not within the limits of 0.8–1.25 except AUC_0-t_ of AAP and MEH in Jelly-S and AUC_0-t_ of AAP in Jelly-H. In addition, the point estimates were remarkably small for AUC_0-t_ and C_max_ of DMH in Tablet-S and Tablet-H. Even though Jelly-S and Jelly-H showed comparable absorption of AAP and MEH, all five formulations were not bioequivalent.

## 4. Discussion

As shown in in vitro dissolution tests in various media, different dissolution profiles resulted in non-bioequivalence in vivo. As expected, the syrup showed the fastest dissolution and absorption compared to the jellies and tablets. The softer jelly exhibited more rapid dissolution than the harder jelly, particularly in pH 4.0, 6.8, and water. However, the difference in the dissolution did not lead to difference in the absorption, suggesting that if the gel strength is 10 N or less, it does not seem to have a significant effect on the bioavailability. In the case of the tablets, the dissolution was the slowest compared to the other three formulations, leading to a decrease in absorption rate and bioavailability. For Tablet-H, which showed disintegration time of about 40 min, the dissolution was delayed much more, and the absorption was also slow and low compared to the syrup. The jellies had less delay in the absorption rate and better bioavailability than the tablets.

The weak correlation between the dissolution and absorption of the four drugs seems to be due to their relatively high solubility and good membrane permeability, suggesting that the dissolution process might not be a rate determining step during the absorption process. Since AAP and CPM are known as BCS class I drugs, formulation factors may not significantly modulate the absorption. In the case of AAP, the absorption was delayed by Tablet-H and the amount of absorption was slightly reduced, but the effect appears to be weak compared to the effect on the other drugs. The absorption of CPM was more delayed and decreased by Tablet-H than AAP. According to several reports, the rate of absorption of CPM is slow probably because the absorption of the weakly basic drug, CPM, occurs mainly in the small intestine and is hardly absorbed in the stomach [11]. DMH has high solubility and permeability throughout the gastrointestinal tract and is reported to be completely absorbed [34]. According to another report by Bolger et al., DMH reaches to the C_max_ in approximately 2.5 h, which is much slower than other typical BCS class I drugs [34]. Bolger et al. demonstrated that dextromethorpan is quickly and extensively metabolized by the gut and liver. In addition, dextromethorphan is a possible lysomotropic drug and lysosomal trapping was identified as the main factor for a slow appearance of the drug in plasma. Based on these facts, the dissolution cannot be an efficient indicator for the systemic absorption because lysosomal tapping is beyond the control of dosage forms [34]. However, the present study shows that DMH in the syrup and jellies reached to the C_max_ in about 1 h. The significant delay in the absorption and lower bioavailability of DMH in Tablet-S and Tablet-H may be due to the slower dissolution and greater exposure to the metabolism in the small intestine and liver. However, this explanation requires additional research. The jellies did not significantly delay the absorption of MEH, but to a significant level by the tablets.

The four formulations were not bioequivalent to the syrup, as they were not equivalent in terms of dissolution. In vitro dissolution studies are sometimes used as an alternative to in vivo studies in assessing bioequivalence of immediate-release solid oral dosage forms containing BCS Class I and III drugs for both cost reduction and ethical considerations [31,35]. Not all generic products containing the same drug in similar strengths and dosage forms are equivalent [31]. Several papers have reported the effects of pharmaceutical excipients on bioavailability of drugs [35]. For example, sorbitol can reduce small intestinal transit time resulting in reduced bioavailability. A few studies have reported that a high dose of sorbitol (≥2.5 g) decreased AUC and C_max_ of ranitidine, metoprolol, and risperidone leading to the failure of bioequivalence [36,37,38]. In the Jelly-S and Jelly-H, 20% of sorbitol (3 g sorbitol in 15 g jelly) was included. Even though there was no significant decrease in bioavailability due to sorbitol in Jelly-S and Jelly-H, the bioequivalence failure might be due to sorbitol. According to Garcia-Arieta et al., their bioequivalence failure was due to a few outliers who were sensitive to the effects of sorbitol on the intestinal transit time [37].

Although the jellies were not bioequivalent to the syrup, it is not believed that this non-equivalence will cause a significant difference in the therapeutic effect of the drug. Old OTC drugs such as the common cold medicines have been used for a long period, and regulatory agencies generally allow manufacturers to formulate ingredients or combinations of ingredients into proprietary products as far as they meet monograph. It has been considered that their therapeutic efficacies are insensitive to the change of formulations. Even so, there should be more research on the relationship between failure in bioequivalence and therapeutic equivalence.

## 5. Conclusions

When the common cold medicines were formulated into different dosage forms, the dissolutions were changed, resulting in the altered bioavailability. According to the criteria by the FDA, the syrup, jellies, and tablets were not bioequivalent. Even so, the jellies and syrup showed similar absorption rates and extents. On the other hand, the tablets significantly delayed and reduced the absorption of the cold medicines compared to the syrup. In conclusion, jelly can be a patient-centered formulation with comparable bioavailability to syrup.

## Figures and Tables

**Table 1:** Composition of oral jellies.

Composition	Jelly-S (gel Strength: 3–4 N)	Jelly-H (gel Strength: 8–9 N)
15 g contains mg of		
Acetaminophen (AAP)	150	150
Chlorpheniramine maleate (CPM)	1.875	1.875
Dextromethorphan hydrobromide (DMH)	11.25	11.25
dl-Methylephedrine hydrochloride (MEH)	18.75	18.75
Carageenan(kappa)	45	45
Carageenan(iota)	60	60
Locust bean gum	60	60
Xanthan gum	-	45
KCl	15	-
Sucralose	15	15
Stevia glucoside	10	10
D-sorbitol	3000	3000
Sodium citrate hydrate	45	45
Citric acid hydrate	18	18
Methyl paraben	1.14	1.14
Propyl paraben	0.285	0.285
Orange flavor	270	270
Purified water	q.s.	q.s.

q.s.: quantum satis.

**Table 2:** Composition of immediate-release (IR) tablets.

Composition	Tablet-S (Hardness: 15 kPa)	Tablet-H (Hardness: 20 kPa)
1 tablet contains mg of		
AAP	150	150
CPM	1.875	1.875
DMH	11.25	11.25
MEH	18.75	18.75
Microcrystalline cellulose (PH-101)	223.125	223.125
Microcrystalline cellulose (PH-102)	37	37
Lactose hydrate	50	50
Low substituted HPC	200	200
HPC	50	50
Colloidal silicon dioxide	50	50
Glyceryl behenate	42	42
Magnesium stearate	8	24
Opadry^®^ (03B28796, White)	26	26

**Table 3:** Drug content in each formulation (% nominal content ± standard deviation).

Drugs	Syrup	Jelly-S	Jelly-H	Tablet-S	Tablet-H
AAP	99.5 ± 0.9	101.5 ± 0.2	98.9 ± 0.4	100.2 ± 0.8	100.8 ± 0.5
CPM	100.3 ± 1.2	100.1 ± 0.9	98.7 ± 0.7	99.8 ± 0.1	99.2 ± 0.5
DMH	99.8 ± 0.3	98.7 ± 0.4	98.7 ± 0.6	99.7 ± 0.4	99.9 ± 0.8
MEH	99.4 ± 0.8	98.2 ± 0.1	99.5 ± 0.6	100.3 ± 1.2	99.5 ± 0.8

**Table 4:** Pharmacokinetic parameters after single oral administration of various formulations (*n* = 15). Each value is expressed as an arithmatic mean ± standard deviation.

Drugs	Parameters	Syrup	Jelly-S	Jelly-H	Tablet-S	Tablet-H
AAP	AUC _0-t_ (µg·h/mL)	12.52 ± 3.01	13.03 ± 4.52	13.22 ± 3.64	11.42 ± 4.27	11.03 ± 4.43
	AUC _0-∞_ (µg·h/mL)	12.59 ± 3.00	13.11 ± 4.57	13.29 ± 3.65	11.55 ± 4.20	11.16 ± 4.34
	C_max_ (µg/mL) *	7.66 ± 2.85	8.48 ± 3.24	8.75 ± 2.90	6.22 ± 2.68	5.38 ± 3.29
	T_max_ (h) ***	0.7 ± 0.5	0.5 ± 0.2	0.5 ± 0.2	1.0 ± 0.3	1.1 ± 0.5
	T_1/2_ (h)	3.1 ± 1.6	3.7 ± 2.6	3.9 ± 3.0	3.5 ± 2.2	3.3 ± 1.6
	MRT (h)	1.6 ± 0.3	1.6 ± 0.4	1.6 ± 0.4	2.2 ± 0.6	2.8 ± 1.5
CPM	AUC 0-t (ng·h/mL)	35.2 ± 18.4	32.1 ± 15.3	32.6 ± 15.6	27.6 ± 15.4	25.0 ± 13.9
	AUC 0-∞ (ng·h/mL)	36.4 ± 18.4	33.8 ± 15.4	34.5 ± 15.5	29.5 ± 14.8	29.9 ± 13.4
	C_max_ (ng/mL)	11.3 ± 6.3	9.4 ± 3.9	9.5 ± 4.9	7.9 ± 4.7	6.9 ± 4.3
	T_max_ (h) **	1.0 ± 0.6	1.4 ± 0.5	1.3 ± 0.5	1.8 ± 0.4	2.5 ± 1.2
	T_1/2_ (h)	2.0 ± 0.3	2.1 ± 0.5	2.0 ± 0.4	2.7 ± 1.9	2.2 ± 0.4
	MRT (h)	3.2 ± 0.5	3.3 ± 0.6	3.3 ± 0.4	4.6 ± 2.5	4.1 ± 0.6
DMH	AUC _0-t_ (ng·h/mL)	14.6 ± 12.6	12.5 ± 10.2	12.0 ± 9.7	8.5 ± 6.2	9.2 ± 7.1
	AUC _0-∞_ (ng·h/mL)	16.0 ± 12.5	13.9 ± 10.8	13.8 ± 9.7	13.3 ± 8.1	12.0 ± 7.9
	C_max_ (ng/mL)	5.6 ± 4.4	3.8 ± 2.5	3.7 ± 3.3	2.6 ± 2.0	2.6 ± 2.5
	T_max_ (h) ***	0.8 ± 0.5	1.2 ± 0.6	1.0 ± 0.4	2.1 ± 1.2	2.3 ± 1.2
	T_1/2_ (h)	2.0 ± 0.4	1.9 ± 0.3	2.0 ± 0.4	2.6 ± 1.6	2.5 ± 1.0
	MRT (h)	3.0 ± 0.6	3.2 ± 0.6	3.4 ± 0.5	4.6 ± 2.2	4.6 ± 1.4
MEH	AUC _0-t_ (ng·h/mL)	127.1 ± 46.2	122.2 ± 37.6	118.6 ± 38.4	116.0 ± 36.8	111.1 ± 47.4
	AUC _0-∞_ (ng·h/mL)	129.0 ± 46.0	124.1 ± 37.5	120.9 ± 38.1	118.9 ± 35.9	115.0 ± 45.5
	C_max_ (ng/mL)	73.7 ± 46.6	62.5 ± 22.2	63.0 ± 27.3	55.0 ± 27.1	45.5 ± 30.9
	T_max_ (h) *	0.7 ± 0.5	0.8 ± 0.4	0.8 ± 0.4	0.9 ± 0.1	1.2 ± 0.5
	T_1/2_ (h)	1.4 ± 0.4	1.3 ± 0.4	1.2 ± 0.3	1.6 ± 0.9	1.7 ± 1.3
	MRT (h)	1.8 ± 0.4	1.9 ± 0.3	1.8 ± 0.2	2.5 ± 1.1	3.0 ± 1.6

Significant at * *p* < 0.1, ** *p* < 0.05, *** *p* < 0.001 after one-way ANOVA with Turkey multiple comparison test for AUC and Cmax, or non-parametric Kruskal–Wallis test followed by Dunn’s multiple comparison. Turkey and Dunn’s post-hoc tests showed no statistical difference between any pairs.

**Table 5:** Point estimates of mean ratio (90% confidence intervals) for AAP.

Test	Parameters	Reference				
		Syrup	Jelly-S	Jelly-H	Tablet-S	Tablet-H
Syrup	AUC_0-t_	-	0.99 (0.86–1.13) ^a^	0.96 (0.83–1.09) ^a^	1.14 (0.99–1.31)	1.19 (1.04–1.37)
	C_max_	-	0.91 (0.68–1.21)	0.85 (0.65–1.15)	1.28 (0.96–1.71)	1.63 (1.22–2.17)
Jelly-S	AUC_0-t_	1.01 (0.88–1.16) ^a^	-	0.97 (0.84–1.11) ^a^	1.16 (1.00–1.33)	1.21 (1.05–1.39)
	C_max_	1.10 (0.83–1.47)	-	0.95 (0.71–1.26)	1.41 (1.06–1.88)	1.80 (1.35–2.39)
Jelly-H	AUC_0-t_	1.05 (0.91–1.20) ^a^	1.03 (0.90–1.18) ^a^	-	1.19 (1.04–1.37)	1.25 (1.09–1.43)
	C_max_	1.16 (0.87–1.55)	1.05 (0.79–1.40)	-	1.49 (1.12–1.98)	1.89 (1.42–2.52)
Tablet-S	AUC_0_-_t_	0.88 (0.76–1.00)	0.86 (0.75–0.99)	0.84 (0.73–0.96)	-	1.04 (0.91–1.20) ^a^
	C_max_	0.78 (0.59–1.04)	0.71 (0.53–0.94)	0.67 (0.50–0.89)	-	1.27 (0.95–1.69)
Tablet-H	AUC_0-t_	0.84 (0.73–0.96)	0.83 (0.72–0.95)	0.80 (0.70–0.92)	0.96 (0.84–1.10) ^a^	-
	C_max_	0.61 (0.46–0.82)	0.56 (0.42–0.74)	0.53 (0.40–0.70)	0.79 (0.59–1.05)	-

^a^ Confidence interval of 90% was within a range of 0.8 to 1.25.

**Table 6:** Point estimates of mean ratio (90% confidence intervals) for CPM.

Test	Parameters	Reference				
		Syrup	Jelly-S	Jelly-H	Tablet-S	Tablet-H
Syrup	AUC_0-t_	-	1.10 (0.92–1.31)	1.06 (0.89–1.26)	1.36 (1.14–1.6)	1.43 (1.20–1.70)
	C_max_	-	1.13 (0.85–1.51)	1.13 (0.85–1.50)	1.54 (1.16–2.06)	1.74 (1.31–2.32)
Jelly-S	AUC_0-t_	0.91 (0.76–1.08)	-	0.96 (0.81–1.15) ^a^	1.23 (1.04–1.47)	1.30 (1.09–1.54)
	C_max_	0.88 (0.66–1.17)	-	1.00 (0.75–1.33)	1.37 (1.03–1.82)	1.54 (1.16–2.05)
Jelly-H	AUC_0-t_	0.94 (0.79–1.12)	1.04 (0.87–1.24) ^a^	-	1.28 (1.07–1.52)	1.35 (1.13–1.60)
	C_max_	0.88 (0.66–1.18)	1.00 (0.75–1.33)	-	1.37 (1.03–1.82)	1.54 (1.16–2.05)
Tablet-S	AUC_0-t_	0.74 (0.62–0.88)	0.81 (0.68–0.97)	0.78 (0.66–0.93)	-	1.05 (0.88–1.25) ^a^
	C_max_	0.65 (0.48–0.86)	0.73 (0.55–0.97)	0.73 (0.55–0.97)	-	1.13 (0.85–1.50)
Tablet-H	AUC_0-t_	0.70 (0.59–0.83)	0.77 (0.65–0.92)	0.74 (0.62–0.88)	0.95 (0.80–1.13) ^a^	-
	C_max_	0.57 (0.43–0.76)	0.65 (0.49–0.87)	0.65 (0.49–0.86)	0.89 (0.67–1.18)	-

^a^ Confidence interval of 90% was within a range of 0.8 to 1.25.

**Table 7:** Point estimates of mean ratio (90% confidence intervals) for DMH.

Test	Parameters	Reference				
		Syrup	Jelly-S	Jelly-H	Tablet-S	Tablet-H
Syrup	AUC_0-t_	-	1.20 (0.94–1.53)	1.17 (0.92–1.49)	1.81 (1.42–2.31)	1.64 (1.29–2.08)
	C_max_	-	1.34 (1.01–1.80)	1.42 (1.06–1.89)	2.22 (1.66–3.00)	2.40 (1.80–3.21)
Jelly-S	AUC_0-t_	0.82 (0.66–1.06)	-	0.97 (0.77–1.24)	1.51 (1.19–1.92)	1.36 (1.07–1.74)
	C_max_	0.74 (0.56–0.99)	-	1.05 (0.79–1.41)	1.65 (1.24–2.21)	1.79 (1.34–2.38)
Jelly-H	AUC_0-t_	0.86 (0.67–1.09)	1.03 (0.81–1.30) ^a^	-	1.55 (1.22–1.97)	1.40 (1.10–1.78)
	C_max_	0.71 (0.53–0.94)	0.95 (0.71–1.27)	-	1.57 (1.17–2.09)	1.69 (1.27–2.26)
Tablet-S	AUC_0-t_	0.55 (0.43–0.70)	0.66 (0.52–0.84)	0.65 (0.51–0.82)	-	0.90 (0.71–1.15)
	C_max_	0.45 (0.34–0.60)	0.61 (0.45–0.81)	0.64 (0.48–0.85)	-	1.08 (0.81–1.44)
Tablet-H	AUC_0_-_t_	0.61 (0.48–0.78)	0.73 (0.58–0.93)	0.71 (0.56–0.91)	1.11 (0.87–1.41)	-
	C_max_	0.42 (0.31–0.56)	0.56 (0.42–0.75)	0.59 (0.44–0.79)	0.93 (0.69–1.24)	-

^a^ Confidence interval of 90% was within a range of 0.8 to 1.25.

**Table 8:** Point estimates of mean ratio (90% confidence intervals) for MEH.

Test	Parameters	Reference				
		Syrup	Jelly-S	Jelly-H	Tablet-S	Tablet-H
Syrup	AUC_0-t_	-	1.03 (0.90–1.17) ^a^	1.05 (0.93–1.20) ^a^	1.08 (0.95–1.23) ^a^	1.16 (1.02–1.32)
	C_max_	-	1.08 (0.83–1.41)	1.09 (0.84–1.43)	0.31 (1.00–1.71)	1.70 (1.30–2.22)
Jelly-S	AUC_0-t_	0.97 (0.86–1.11) ^a^	-	1.03 (0.90–1.17) ^a^	1.06 (0.93–1.20) ^a^	1.13 (0.99–1.29)
	C_max_	0.93 (0.71–1.21)	-	1.01 (0.77–1.32)	1.21 (0.93–1.58)	1.57 (1.20–2.05)
Jelly-H	AUC_0-t_	0.95 (0.83–1.08) ^a^	0.97 (0.85–1.11) ^a^	-	1.03 (0.90–1.17) ^a^	1.10 (0.97–1.25) ^a^
	C_max_	0.91 (0.70–1.19)	0.99 (0.76–1.29)	-	1.20 (0.92–1.56)	1.55 (1.19–2.03)
Tablet-S	AUC_0-t_	0.92 (0.81–1.05) ^a^	0.95 (0.83–1.08) ^a^	0.97 (0.86–1.11) ^a^	-	1.07 (0.94–1.22) ^a^
	C_max_	0.76 (0.59–1.00)	0.83 (0.63–1.08)	0.84 (0.64–1.09)	-	1.30 (0.99–1.70)
Tablet-H	AUC_0-t_	0.86 (0.81–1.05) ^a^	0.88 (0.78–1.00)	0.91 (0.80–1.04) ^a^	0.94 (0.82–1.06) ^a^	-
	C_max_	0.59 (0.45–0.77)	0.64 (0.49–0.83)	0.64 (0.49–0.84)	0.77 (0.59–1.00)	-

^a^ Confidence interval of 90% was within a range of 0.8 to 1.25.
